# Unsupervised multimodal modeling of cognitive and brain health trajectories for early dementia prediction

**DOI:** 10.1038/s41598-024-60914-w

**Published:** 2024-05-10

**Authors:** Michael C. Burkhart, Liz Y. Lee, Delshad Vaghari, An Qi Toh, Eddie Chong, Christopher Chen, Peter Tiňo, Zoe Kourtzi

**Affiliations:** 1 Department of Psychology, University of Cambridge, Cambridge, CB2 3EB UK; 2 Department of Pharmacology, Memory, Aging, and Cognition Center, Yong Loo Lin School of Medicine, National University of Singapore, Singapore, Singapore; 3 School of Computer Science, University of Birmingham, Birmingham, B15 2TT UK

**Keywords:** Biomarkers, Dementia, Computational science, Cognitive ageing

## Abstract

Predicting the course of neurodegenerative disorders early has potential to greatly improve clinical management and patient outcomes. A key challenge for early prediction in real-world clinical settings is the lack of labeled data (i.e., clinical diagnosis). In contrast to supervised classification approaches that require labeled data, we propose an unsupervised multimodal trajectory modeling (MTM) approach based on a mixture of state space models that captures changes in longitudinal data (i.e., trajectories) and stratifies individuals without using clinical diagnosis for model training. MTM learns the relationship between states comprising expensive, invasive biomarkers (β-amyloid, grey matter density) and readily obtainable cognitive observations. MTM training on trajectories stratifies individuals into clinically meaningful clusters more reliably than MTM training on baseline data alone and is robust to missing data (i.e., cognitive data alone or single assessments). Extracting an individualized cognitive health index (i.e., MTM-derived cluster membership index) allows us to predict progression to AD more precisely than standard clinical assessments (i.e., cognitive tests or MRI scans alone). Importantly, MTM generalizes successfully from research cohort to real-world clinical data from memory clinic patients with missing data, enhancing the clinical utility of our approach. Thus, our multimodal trajectory modeling approach provides a cost-effective and non-invasive tool for early dementia prediction without labeled data (i.e., clinical diagnosis) with strong potential for translation to clinical practice.

## Introduction

Dementia due to Alzheimer’s disease (AD) involves a cascade of pathophysiological processes from normal cognition to Mild Cognitive Impairment (MCI) to dementia, with different markers of progression across disease stages^1,2^. Despite decades of research and development, clinical trials of potential disease-modifying treatments for dementia have remained largely unsuccessful. However, recent developments in drug discovery (e.g.^3,4^) call for interventions earlier in the progression of disease^5,6^ to enhance patient outcomes and aid future clinical trials. Identifying individuals at-risk and predicting dementia early (i.e., at early disease stages or before the onset of symptoms) have strong potential to impact clinical management, drug discovery, and treatment outcomes. Yet, early dementia prediction remains challenging in the following key respects.

First, neuroimaging-derived biomarkers (i.e., MRI, PET) have been shown to be important for detecting neurodegeneration. For Alzheimer’s Disease (AD) in particular, there is a strong link between biomarkers (i.e., β-amyloid accumulation, tau accumulation, neurodegeneration) and symptoms (i.e., cognitive decline)^1,2,7,8^. However, PET scans that use radioactive contrast agents to extract biomarkers (β-amyloid, tau) are invasive and expensive (e.g., over $3000 for an Amyloid PET scan in the USA^9^) for large-scale use in the general population, resulting in health inequalities related to their availability across healthcare settings. Addressing this challenge raises the need for early prediction from low-cost and non-invasive measures (e.g., cognitive tests alone). Second, predicting dementia at early or pre-symptomatic stages of the disease means that individuals have not yet been assigned a clinical diagnosis. Recent work on machine learning and mathematical modeling for dementia prediction predominantly focusses on supervised models (e.g., SVMs, neural networks) using cross-sectional labelled data from patients that have clinical diagnoses (see, e.g.,^10–12^, for recent reviews). In contrast, making predictions based on unlabeled data requires unsupervised modeling approaches. Third, the current diagnostic framework suffers from misdiagnosis (estimated sensitivity 70.9–87.3%; specificity: 44.3–70.8%)^13,14^ in the early stages of Mild Cognitive Impairment (MCI), potentially due to age-related comorbidities (e.g., geriatric depression, stroke) that result in cognitive decline^15,16^. Modeling approaches that make predictions based on binary clinical labels risk incorporating this misdiagnosis into their predictions. Thus, novel trajectory modeling approaches are needed to reduce misdiagnosis at early stages by capturing changes in biomarkers and cognition based on longitudinal data rather than classifying patients based on clinical diagnosis.

**Figure 1:**
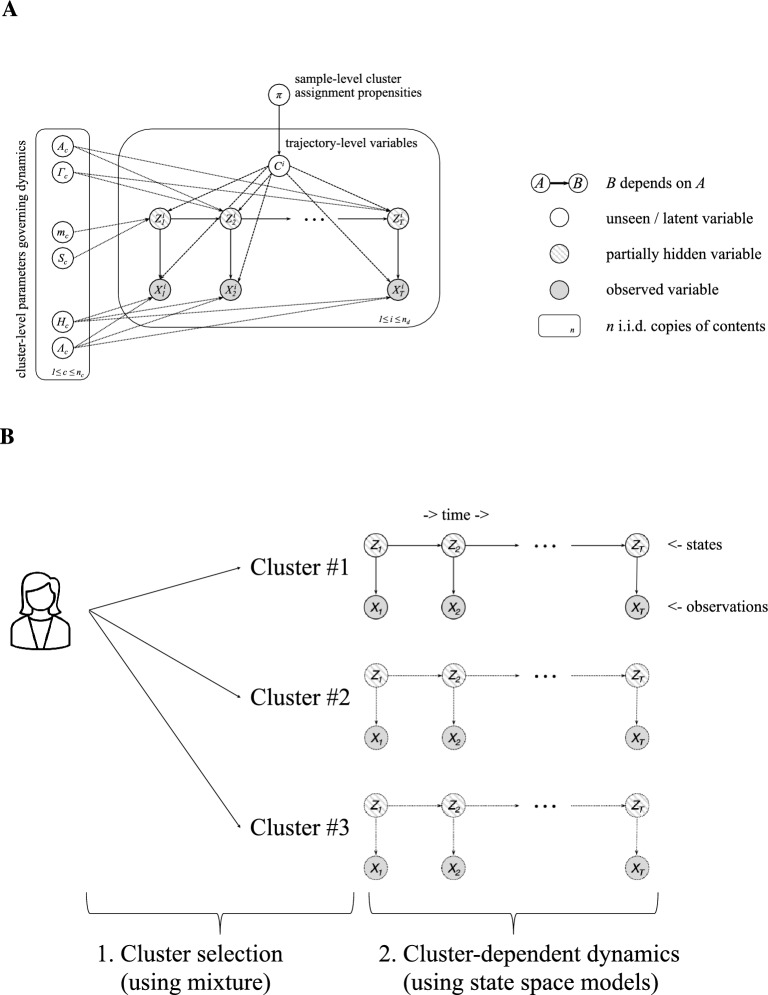
Modeling approach. (**A**) Plate notation for the mixture of state space models. In this directed acyclic graph, each node represents a variable. Any two variables are independent after conditioning on their parents. Full shading denotes variables available during both training and testing, half shading denotes variables available during training but not necessarily during testing, and the remaining variables with no shading must be inferred from data. Plates indicate repetition. The plate on the left corresponds to the cluster-specific parameters for each of the $n_c$ clusters. The larger plate governs the trajectory of each of the $n_d$ samples. (**B**) Generative modeling process. The model independently assigns each individual to a cluster and then generates a sequence of states with corresponding observations according to the cluster-specific dynamics for the assigned cluster.

**Figure 2:**
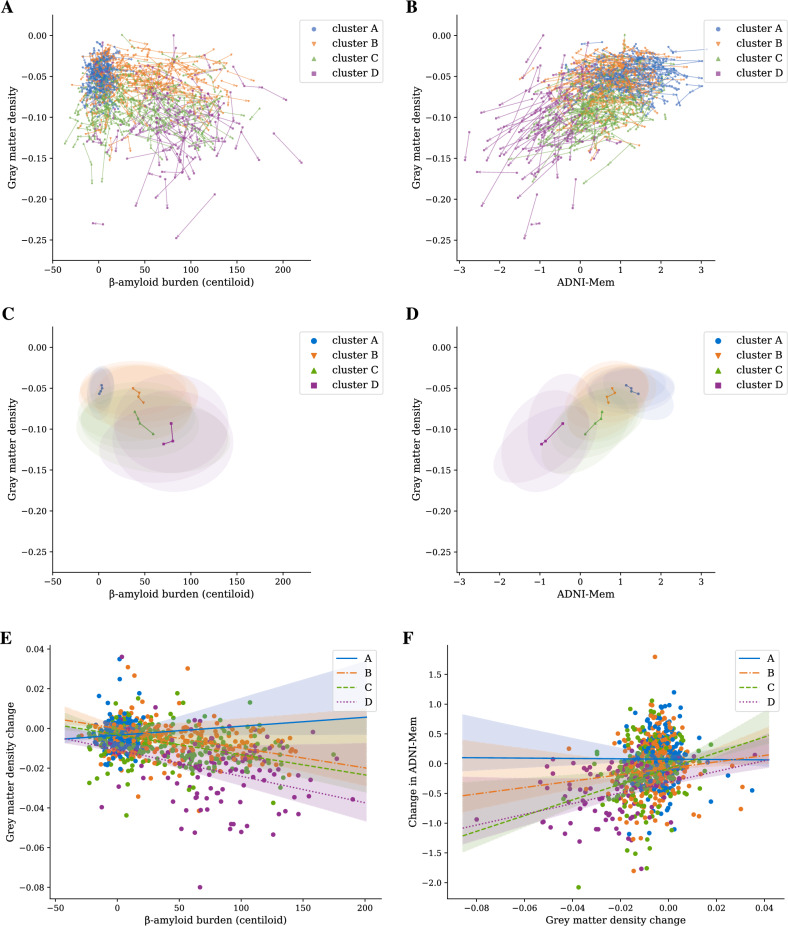
4-cluster model trained on ADNI data. (**A**) Progression of states over time illustrating the relationship between state variables (grey matter density, β-amyloid burden in centiloid), colored by cluster. (**B**) Progression of states over time illustrating the relationship between a state (grey matter density) and an observed (ADNI-Mem) variable. (**C**) Average trajectories per cluster in the state variables (grey matter density, β-amyloid burden in centiloid) weighted by cluster assignment probability. For each cluster at each point in time, shading denotes a confidence region from a gaussian with mean and covariance corresponding to the empirical mean and covariance of trajectories with available data, weighted according to cluster assignment probability. For cluster D, the final time step contains only 7 observations, and so is not plotted. (**D**) Average trajectories per cluster for grey matter density and ADNI-Mem weighted by cluster assignment probability. Shading as in C. Final time step in cluster D is not plotted to make a fair comparison with C. (**E**) Relationship of changes in grey matter density over a 2-year period to β-amyloid burden per cluster. Lines depict cluster-specific slopes and intercepts learned from a linear mixed effects model. Shaded areas depict a 95% confidence interval for each line. (**F**) Relationship of changes in ADNI-Mem (over a 2-year period) to changes in grey matter density (over a 2 year-period). Lines and shading same as for E.

**Figure 3:**
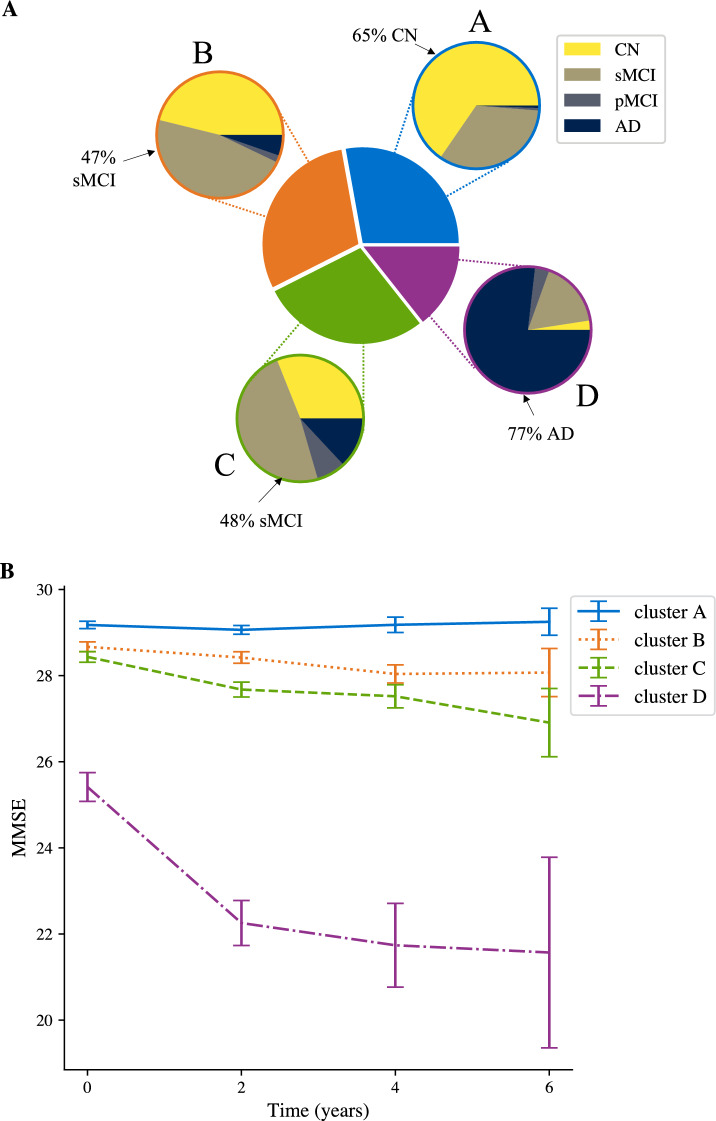
Profiling ADNI clusters. (**A**) Pie charts indicating cluster assignment and break down into clinical diagnosis, indicating the probability of a clinical outcome given cluster assignment. (**B**) Average MMSE (±1 std. error) per cluster over time. Note that years 4 and 6 only include trajectories of length at least 3 and 4, respectively (i.e. year 4: $n = 194$; year 6, $n= 40$).

To tackle these key challenges for early dementia prediction, we propose an unsupervised multimodal trajectory modeling (MTM) approach based on a mixture of state space models that trains on longitudinal data (states, observations) to learn relationships between the progression of neuroimaging-derived biomarkers and cognitive data and stratifies individual trajectories into clusters of cognitive health (Fig. 1). States correspond to neuroimaging-based biomarkers (β-amyloid, grey matter density): these features are available during model training but may be unavailable or missing when the model is tested (a scenario known in machine learning as Learning with Privileged Information^17^). Observations correspond to cognitive test scores that can be collected inexpensively and non-invasively at scale and have been shown to relate to biomarker levels^18–20^. Our modeling assumptions allow cluster formation to be driven by the biomarker dynamics that characterize the progression of AD, with cognitive measurements tracking biomarker values. We train the MTM on longitudinal data from the Alzheimer’s Disease Neuroimaging Initiative (ADNI;^21^) and we derive clusters that stratify individuals (from independent test data) based on cognitive decline and clinical outcomes (i.e., clinical diagnosis) that have not been used in model training. Further, we demonstrate that the trained multimodal trajectory model stratifies individuals into discriminable clusters when tested with cognitive data alone (i.e., observations without access to biomarkers) or a single assessment (rather than longitudinal data). Importantly, MTM allows us to derive an individualized cognitive health index based on cluster membership that predicts conversion to AD more precisely than standard clinical data (grey matter atrophy, β-amyloid, cognitive scores). Finally, we provide out-of-sample validation by testing the trained MTM (i.e., training on research cohort data: ADNI)-on real-world patient data from memory clinics (Memory Ageing & Cognition Centre at the National University of Singapore: MACC). MTM generalizes successfully, despite missing data, stratifying MACC patients into distinct clusters, as profiled by clinical diagnoses and clinical scales (i.e., mini-mental state examination, MMSE^22^). These findings provide evidence for a robust unsupervised trajectory modeling approach that delivers early prediction on unlabeled multimodal data, handles missing data, and generalizes from research to real-world patient data, enhancing the clinical utility of our modeling approach and its potential for translation to clinical practice.

## Methods

### ADNI data

Data for this study comprised 571 trajectories from the Alzheimer’s Disease Neuroimaging Initiative (ADNI; Table S1) database comprising 2–4 assessments (trajectory length $= 2$, $n= 337$; trajectory length $= 3$, $n= 194$; trajectory length $= 4$, $n= 40$). Trajectories used for model training comprised longitudinal measurements of neuroimaging-based biomarkers (grey matter density from medial temporal cortex^20^ and Florbetapir-based amyloid score^23^ temporally aligned (within 6 months) with cognitive test scores (ADNI-Mem for memory^24^, ADNI-EF for executive functioning^25^, the Montreal Cognitive Assessment (MoCA)^26^, and the Alzheimer’s Disease Assessment Scale (ADAS-13))^27^. To profile the clusters, we used clinical diagnosis and cognitive decline as measured with longitudinal scores from the mini-mental state examination (MMSE^22^) that is commonly used in clinical practice. Clinical diagnoses based on individuals’ final assessments were as follows: cognitively normal (CN: 234), stable MCI (sMCI: 224), progressive MCI (pMCI: 19), and Alzheimer’s disease (AD: 94). Patients were identified as pMCI if they progressed from MCI to AD in a 3-year period while sMCI patients remained diagnosed as MCI for the same period.

### MACC data

We tested MTM on independent memory clinic cohort data from the Memory Ageing & Cognition Centre at the National University of Singapore (MACC; Table S1). The MACC dataset comprises 158 trajectories (trajectory length = 2, $n = 21$; trajectory length = 3, $n = 137$) containing a single β-amyloid PET measurement and at least one measurement of grey matter (GM) density score derived from structural MRI scans (1 GM score, $n=5$; 2 GM scores, $n=52$; 3 GM scores, $n=101$). MoCA and MMSE scores were available for most patients. However, ADNI-Mem, ADNI-EF, and ADAS-13 that were used for training the MTM on ADNI data were not available in the MACC dataset. Clinical outcomes based on individuals’ final assessments were as follows: cognitively normal (CN, $n=36$), mild MCI ($n=50$), moderate MCI ($n=18$), and Alzheimer’s disease (AD, $n= 54$).

## Modeling approach

*Unsupervised generative model*: as neurodegeneration progresses heterogeneously, we learn a probabilistic mixture of trajectory models on longitudinal data. Each prototypical mixture component takes the form of a state space model where biomarkers are states and cognitive test scores are observations. Explicitly, we model the sequences $z^i_{1:T} = (z_1^i,\dotsc ,z_T^i)$ of biomarkers and $x^i_{1:T} = (x_1^i,\dotsc ,x_T^i)$ cognitive test scores for the *i*th participant ($1\le i \le n_d$) as an i.i.d. sample from

1 $$\begin{aligned} p(z^i_{1:T}, x^i_{1:T}) = \textstyle \sum _{c=1}^{n_c} \pi _{c} \delta _{\{c=c^i\}} \cdot p(x^i_{1:T}; z^i_{1:T}|c^i) \end{aligned}$$

where $c^i \in \{1,\dotsc ,n_c\}$ denotes the assigned cluster, $\delta _{\{c=c^i\}}$ is 1 when $c=c^i$ and 0 otherwise, and $p(c)=\pi _c$ is a categorical distribution on the $n_c$ clusters. For each cluster $1\le c\le n_c$, we specify a linear, Gaussian model

2 $$\begin{aligned} p(x^i_{1:T}; z^i_{1:T}|c) = \textstyle \eta _d(z_1; m_c, S_c) \prod _{t=2}^T \eta _d(z_t;z_{t-1}A_c,\Gamma _c) \prod _{t=1}^T \eta _\ell (x_t;z_tH_c,\Lambda _c), \end{aligned}$$

where $m_c$ and $S_c$ correspond to the mean and covariance of the first biomarker measurement, $A_c$ and $\Gamma _c$ govern the dynamics of the biomarkers, and $H_c$ and $\Lambda _c$ describe the relationship between the biomarkers and cognitive scores. In an abuse of notation, we allow *T* to depend on *i*; our framework allows trajectories to have differing lengths. Figure 1a contains our full modeling framework in plate notation. In contrast to the pioneering work of Chiappa and Barber who fit models using variational inference with probabilistic assignment^28^, we fit models using expectation–maximization (EM)^29^ with hard latent indicator variable assignment in the E-step. This affords us the possibility of readily extending our framework to allow nonlinear relationships between biomarkers and cognitive scores. To avoid local optima, we train models from multiple different random initializations and select the one with the highest objective on training data.

Each mixture component is characterized by the underlying dynamics of the biomarkers and the relationship between biomarkers and cognitive scores (Fig. 1a). In the proposed framework, the first-order Markovian sequence of biomarker levels drives our process of interest and the cognitive scores at each assessment can be viewed as read-outs of a patient’s biomarker levels at a given time/disease stage. That is, as the underlying biological dynamics closely captured by changes in biological markers (i.e. grey matter density, β-amyloid) drive cognitive decline, the current state of cognitive decline can be then non-invasively “read-out” from the system in terms of cognitive scores. In this context, cognitive scores could be interpreted as causally driven by changes in biological markers. Training the multimodal trajectory model requires access to trajectories that include both biomarkers and cognitive scores, in contrast to previous work that inferred underlying states^30,31^. Giving MTM access to states during training leads to a simpler and more straightforward process for inferring model parameters. Following training, marginalizing over missing variables allows us to stratify new trajectories that have missing biomarkers or incomplete cognitive scores (Supplementary Information).

*Model selection:* To choose the optimal number of components for the MTM, we calculate the Bayesian information criterion (BIC)^32^ versus the number of clusters.

*Cross-validation:* To assess stability of cluster assignment to variations in training data, we performed 10-fold cross validation on the ADNI dataset as follows. For each fold, we trained the MTM using the data from the 9 other folds ($n\approx 514$ trajectories) and tested on the remaining data ($n\approx 57$ trajectories). This gives us 10 models, but only one model is used to make a prediction for any given datapoint (i.e., the unique model that did not include the datapoint in its training set). The prediction from this model is the unique cross-validated prediction. Performing cross-validation with unsupervised models entails an additional challenge of harmonizing the labels across the ten separate mixture models learned during training. To harmonize across folds, we assigned labels to clusters alphabetically (i.e. cluster A–D) with A corresponding to the cluster with the lowest percentage of individuals with an AD diagnosis in the training set, B to the cluster with the second lowest percentage, C to the cluster with the third lowest percentage, and D to the cluster with the fourth lowest (i.e. highest) percentage of individuals with an AD diagnosis in the training set. Table S2 shows concordance in the percentage of individuals with an AD diagnosis per cluster across the 10 cross-validation folds. Further, for each trajectory assigned to a given cluster during testing, we determine the number of training runs that assign the trajectory to that cluster. Note that for 10-fold cross-validation, each trajectory is in a test set once and in a training set 9 times, resulting in values ranging from 0 to 9. Fig. S1 shows that the histograms concentrate on higher values (ranging from 7 to 9), indicating that most individual trajectories remain in the same cluster across cross-validation runs.

*Model-derived index:* Given a fitted model, we define the cognitive health index (up to an additive constant) as the logarithm of the likelihood under the cluster A model. This is given explicitly by:

$$\begin{aligned} M(z^i_{1:T}, x^i_{1:T}) = \log (p(x^i_{1:T}; z^i_{1:T}|c^i = A)). \end{aligned}$$

We used cross-validation to evaluate the model-derived index, in the same manner as we used to make predictions. This prevents information leakage, as the index is always calculated on data not used for model training.

## Results

### MTM stratifies individuals based on health trajectories

Our multimodal trajectory model (MTM) effectively stratifies individuals to clusters of cognitive health based on the relationship between longitudinal biomarker (β-amyloid, grey matter density) and cognitive data (ADNI-Mem, ADNI-EF, MoCA, ADAS-13). Informed by both BIC (Bayesian information criterion) and an elbow plot (Fig. S2), we present results for a 4-cluster MTM trained and tested on ADNI data in an unsupervised manner (i.e. without labelled data). Figure 2A, C shows 4 trajectory clusters based on the relationship of state variables (i.e. biomarkers: grey matter density vs. β-amyloid) derived from the model following 10-fold cross validation. Figure 2B, D shows 4 trajectory clusters based on the relationship of state (grey matter density) and cognitive (ADNI-Mem) data. In particular, individuals in different clusters vary in disease progression based on the combination of different markers (Fig. S3, Table S3 for nonlinear dynamics). Individuals in cluster A show the highest grey matter density, lowest β-amyloid burden and highest cognitive scores, while individuals in cluster D show the highest β-amyloid burden, lowest grey matter density and lowest cognitive scores with clusters B and C falling between A and D (Fig. 2C, D). That is, individuals in cluster C have similar β-amyloid accumulation but higher grey matter atrophy compared to individuals in cluster B, suggesting a higher degree of neurodegeneration.

Next, we demonstrate that MTM-derived clusters of cognitive health differ in biomarker dynamics (Fig. 2E) and the relationship between biomarker dynamics and cognitive decline (Fig. 2F) over time. In particular, we used a linear mixed effects model (LME) to test whether β-amyloid burden predicts change in grey matter density over time. This LME analysis showed a significant relationship between biomarkers (main effect of cluster $F_{3, 585}=12.0, p<0.001$, interaction $F_{4, 625}=11.4, p<0.001$). The estimated cluster-specific trends (slopes for a linear approximation of grey matter density change versus β-amyloid burden) decreased monotonically by cluster and differed significantly from zero for all clusters (B: $t_{592}=-3.62, p<0.001$; C: $t_{574}=-3.83, p<0.001$; D: $t_{667}=-4.18, p<0.001$) except cluster A ($t_{681}=0.62, p=0.534$). Similarly, testing (linear mixed effects model) whether changes in grey matter density predict changes in cognition (ADNI-Mem) showed a significant main effect of cluster ($F_{3, 837}=6.1, p<0.001$) and interaction ($F_{4, 837}=8.1, p<0.001$). The estimated cluster-specific trends were significantly different from zero for clusters C & D (C: $t_{837}=4.18, p<0.001$, D: $t_{837}=3.53, p<0.001$) but not A or B (A: $t_{837}=-0.07, p=0.94$, B: $t_{837}=1.59, p=0.11$).

To enhance MTM interpretability, we profiled MTM-derived clusters using clinical diagnoses (Fig. 3A & S4A, Table S4). 98% of individuals in cluster A and 93% in cluster B have a cognitively normal (CN) or sMCI diagnosis, whereas 21% of individuals in cluster C and 81% in cluster D have an AD or pMCI diagnosis. Conversely, 78% of individuals diagnosed as cognitively normal are assigned to clusters A or B, while 89% of individuals diagnosed as AD are assigned to clusters C or D. These clusters differed significantly in their observed frequencies of clinical outcomes ($\chi ^2(9)=308.01, p<0.001$). Employing a 2-cluster model results in less precise stratification than 4 clusters, showing a coarser partition of our sample; that is, 94% of individuals in the first cluster are diagnosed as CN or sMCI (2% pMCI, 3% AD), while 58% of individuals in the second cluster are diagnosed as AD (8% CN, 27% sMCI, 7% pMCI).

We next profiled clusters by rate of cognitive decline, as indicated by age-adjusted MMSE, a scale typically used in clinical practice (Fig. 3B; note MMSE was not included in model training). A linear mixed effects model used to predict age-adjusted MMSE showed significant effects for cluster, time, and a significant interaction between cluster and time (main effect of cluster $F_{3, 874} =61.8, p<0.001$; main effect of time $F_{1, 932}=157.1; p<0.001$; interaction: cluster x time $F_{3, 929}=64.1, p<0.001$). The estimated trends for clusters A and B were not significantly different from zero ($A: t_{922}=0.810, p=0.42; B: t_{923}=-1.74, p=0.082$), while trends for clusters C and D were significantly lower than zero ($C: t_{922}=-3.80, p<0.001; D: t_{938}=-13.2, p<0.001$). The trend for cluster D was significantly lower than the trends for clusters A, B, C (post-hoc comparisons, A vs. D, $t_{944}=13.2, p<0.001$; B vs. D, $t_{946}=12.2, p<0.001$; C vs. D, $t_{945}=10.4, p<0.001$). These results suggest that individuals in clusters A and B may show a slower cognitive decline consistent with normal aging, in contrast to individuals in cluster D that decline at a rate higher than 1 point of MMSE per year (i.e., 95% confidence interval for trend of D: $[-1.35, -1.05]$).

**Figure 4:**
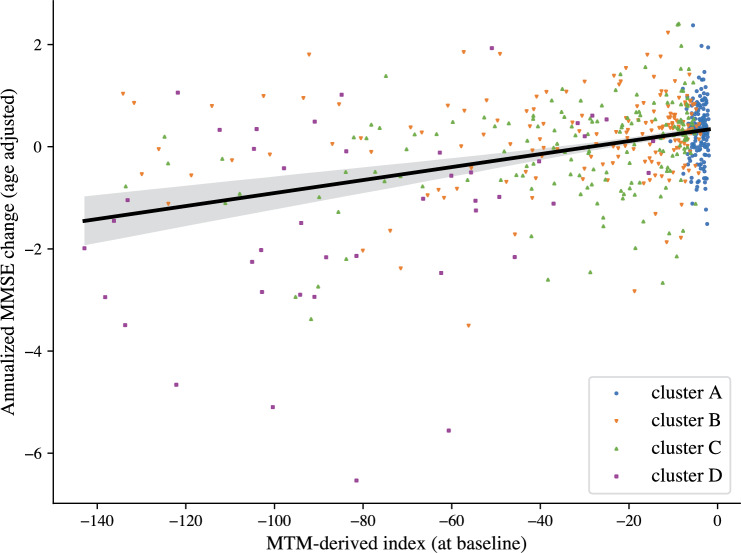
Prognostic MTM-derived index relates to cognitive decline. Correlation of MTM- derived index calculated using baseline data with age-adjusted annualized MMSE change, indicating that the model-derived index relates to cognitive decline (Pearson’s $r=0.396; p<0.001$). Outliers in the MTM-derived index (values lower than 3 standard deviations from the mean; 2% of the sample) were excluded for illustration purposes. Points colored according to cluster assignment from baseline data alone.

**Figure 5:**
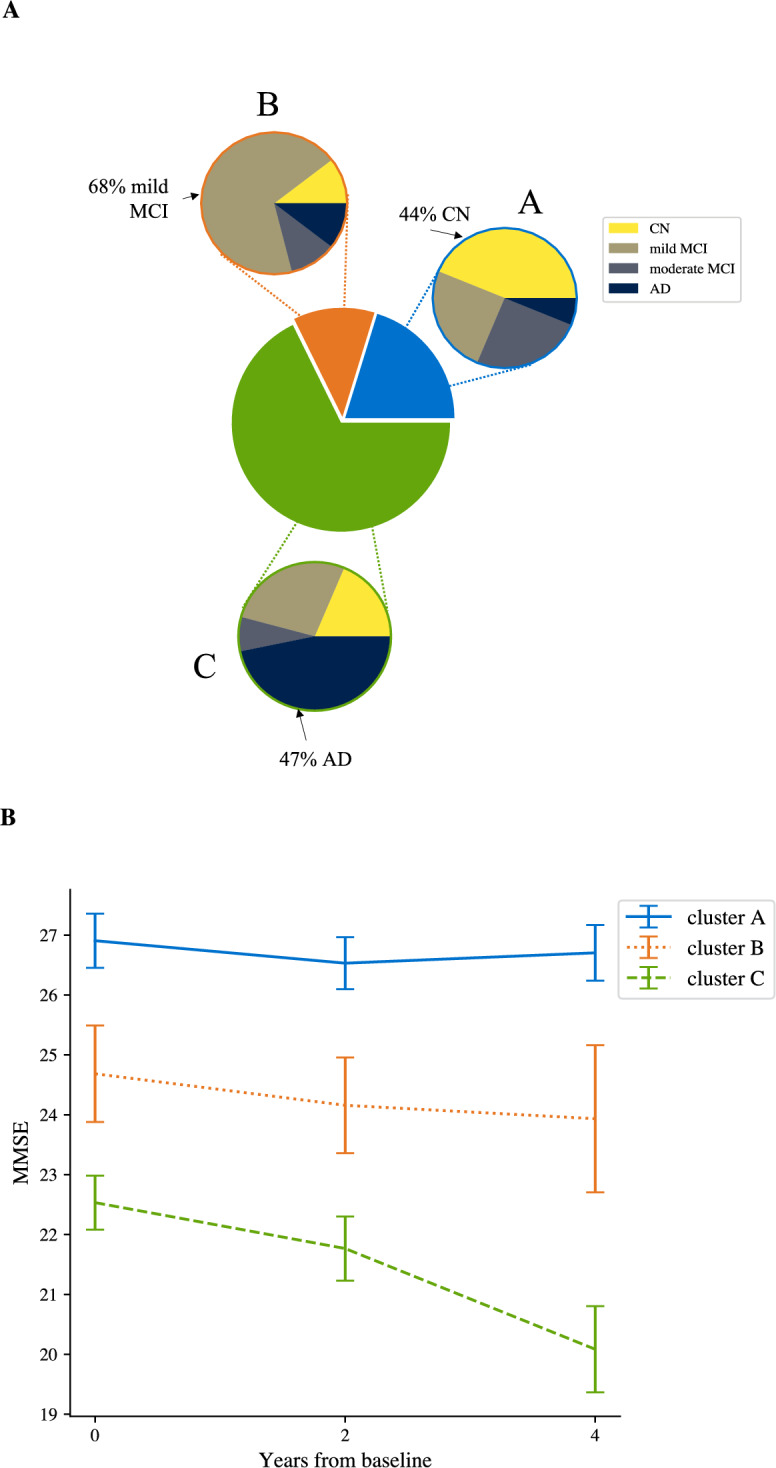
Transfer to clinical data: Profiling MACC clusters. (**A**) Pie charts indicating cluster assignment and break down into clinical diagnosis indicating the frequency of each clinical diagnosis given cluster assignment. (**B**) Average MMSE (±1 std. err.) per cluster over time.

### MTM-derived cognitive health index predicts conversion to AD

We next tested whether assignment of individuals to MTM-derived clusters allows us to make predictions about progression to AD. To quantify individual membership to MTM-derived clusters, we derived a cognitive health index as the logarithm of the likelihood under the cluster A model. We showed that this MTM-derived index relates to future cognitive decline as indicated by age adjusted rate of MMSE change/year (Pearson’s, A = 0.396, p < 0.001, Fig. 4). Further, using logistic regression, we showed that this MTM-derived index predicts conversion to AD from baseline data. ROC analysis showed higher AUC for the MTM-derived index (0.878) than a) univariate markers: MMSE (0.812), β-amyloid (0.804), GM density (0.736), b) combinations of markers: GM density and β-amyloid (AUC = 0.850), GM density and MMSE (AUC = 0.852). We corroborated these results by training Cox proportional hazard models to predict conversion to AD from baseline data. Concordance as measured with cross-validation was higher for the MTM-derived index (0.836) than β-amyloid (0.807), GM density (0.703) or cognitive scores (e.g. ADNI-mem: 0.829, MOCA: 0.801). These results suggest that the MTM-derived cognitive health index predicts conversion to AD more precisely than commonly-used clinical assessments.

### MTM training on trajectories vs. baseline data alone

We asked whether training MTM on longitudinal data (i.e. trajectories) provides an advantage over training on baseline data alone. We compared the 4-component MTM model trained on longitudinal data to a 4-cluster Gaussian mixture model trained on baseline data alone (GMM-baseline) using the same cross-validation approach as for MTM. We found only 52.9% agreement between the cluster labels assigned by GMM-baseline vs. MTM. Further, comparing distributions of clinical diagnoses across clusters showed that clusters derived by GMM-baseline ($\chi ^2(9) = 226.4, p< 0.001$) have a more uniform distribution of clinical diagnoses than MTM-derived clusters ($\chi ^2(9) = 308.0, p < 0.001$). Comparing between models (Table S5) showed significantly higher deviation of the joint distribution of cluster assignment and clinical diagnosis from the product of the marginals for MTM than GMM-baseline (Sharma-Song test for second-order differentials in contingency tables^33^, $\chi ^2 (9) = 48.3, p< 0.001$). These results suggest that MTM training on longitudinal rather than baseline data alone stratifies individuals more reliably to clusters of cognitive health based on future disease progression.

### MTM stratification based on a single assessment

Collecting longitudinal data often proves challenging and costly in clinical practice, while multiple visits lengthen waiting time to diagnosis for patients. Here, we test whether the MTM trained on longitudinal data stratifies individuals reliably when tested using a single assessment alone. In particular, we tested the MTM on the first (i.e., baseline) or final assessment available per individual in an independent dataset.

We found that MTM maintains similar stratification (as profiled by clinical diagnosis) when tested on a single assessment. 81% of individuals remained in the same cluster when using the initial assessment data compared to trajectory data; this increased to 85% for the final assessment (columns III–VI in Table S4). These results strengthen the clinical utility of our modeling approach, suggesting that MTM stratifies individuals reliably based on single patient assessments (e.g., first baseline assessment) with potential impact in expediting diagnosis and improving clinical management pathways.

### MTM stratification based on cognitive data alone

We next asked whether MTM stratifies individuals reliably when tested with low-cost non-invasive data (i.e., cognitive observations) in the absence of state variables comprising biomarkers. We tested the MTM—trained on both biomarker and cognitive data—on held-out test data (during the cross-validation process) comprising cognitive scores alone (i.e., ADNI-Mem, ADNI-EF, MoCA, and ADAS-13). We show that the model clusters individuals to discriminable trajectories of cognitive health and partly maintains the stratification we observed when testing with both biomarker and cognitive data (Table S4: columns II, IV, & VI). That is, 58% of individuals remained in the same MTM-derived cluster when MTM was tested with cognitive data alone, while 26% individuals were stratified to a cluster with lower cognitive decline compared to MTM stratification based on both biomarker and cognitive data. These results suggest that MTM handles missing biomarker data and stratifies individuals based on non-invasive data (e.g., cognitive assessments), enhancing the clinical utility of our modeling approach.

### Out of sample validation and model transfer from research to clinical data

To test the generalizability and validate the clinical utility of our approach, we tested the trained MTM (i.e. training on ADNI data), on independent memory clinic cohort data from the Memory Ageing & Cognition Centre at the National University of Singapore (MACC). This is a challenging task that requires transfer from a research to a clinical dataset with missing data; that is, trajectories ($n=158$) in the MACC dataset comprise single β-amyloid PET measurements, some longitudinal structural MRI scans, and limited cognitive data (MoCA and MMSE for most participants but no ADNI-Mem, ADNI-EF, and ADAS-13 data that were used for training the MTM).

MTM stratifies patients to clinically meaningful clusters when (I) using trajectory data, (II) cognitive scores alone (i.e., MoCA), or (III) data from the final assessment alone (Table S6). A 3-cluster MTM (as determined by BIC; Fig. S2) showed that 94% of individuals assigned to cluster A have CN or MCI diagnosis, whereas 47% of individuals assigned to cluster C have AD diagnosis (Fig. 5A). Conversely, 74.4% of individuals diagnosed as cognitively normal (CN) are assigned in clusters A or B and 73.6% of individuals diagnosed as AD are assigned to cluster C (Fig. S4B). These MTM-derived clusters differ significantly in their observed frequencies of clinical outcomes ($\chi ^2(6) = 39.5, p <0.001$).

Profiling the clusters for cognitive decline showed significant differences in rate of cognitive decline (i.e. rate of MMSE change/year) between clusters A and C (Fig. 5B; linear mixed effects model of MMSE over time including age). A mixed effects model for age-adjusted MMSE showed a significant effect for cluster ($F_{2, 195.11}=10.8, p<0.001$) and a significant cluster x time interaction ($F_{3, 294.97}=9.23, p<0.001$). We found that the estimated trends for clusters A and B were not significantly different from zero (A: $t_{295}=0.527, p=0.60$; B: $t_{296}=-0.433, p=0.66$), while for cluster C the trend was significantly below zero ($t_{294}=-5.22, p <0.001$). The trend for cluster C was significantly higher than for cluster A ($t_{295}=2.93, p=0.010$), suggesting that individuals in cluster C are predicted to have higher rate of cognitive decline.

Finally, stratification in discrete clusters was maintained when testing the MTM using limited data (i.e. first assessment or cognitive data alone). That is, 79% of individuals remained in the same cluster when MTM was tested with data from the first assessment. When MTM was tested with limited cognitive data (MoCA alone) without biological (i.e. GM density) data, 48% of individuals retained their cluster assignment. 44% of individuals were assigned to a healthier cluster, suggesting that when biomarker data is missing, the model assigns more individuals to cluster A, corresponding to a healthier trajectory. This is consistent with MTM stratification on ADNI data, suggesting that biomarker data may provide additional information for disease progression.

## Discussion

We develop and validate an unsupervised multimodal trajectory modeling approach (MTM) that stratifies individuals early and precisely based on their brain and cognitive health trajectories. MTM learns class-specific relationships over time between biomarkers-that are acquired through costly and invasive measurements (e.g., PET scans)-and cognitive observations that are acquired through less costly and non-invasive testing. To tackle the challenge of early dementia prediction with unlabeled data (i.e. at early or pre-symptomatic disease stages before cognitively normal individuals have a clinical diagnosis), we adopt an unsupervised training approach. In contrast to most machine learning models for dementia prediction that adopt supervised learning to classify patients based on clinical diagnosis, our unsupervised MTM approach stratifies individuals based on their brain and cognitive health trajectories without using clinical labels for model training.

Working on a generatively specified framework allows us to obtain interpretable models of prototypical biomarker and cognitive progression, handle trajectories of variable lengths, short observation sequences (i.e. single assessments), and missing data (i.e. cognitive data alone). Our modeling approach is inspired by recent work on unsupervised trajectory clustering for AD prediction including mixtures of generalized mixed effects models^34^, hierarchical mixture models of longitudinal Siamese neural networks^35^, and mixtures of Gaussian processes inductively biased towards monotonic decline^36^. Ramamoorthy^36^ found that the majority of the largest AD-related trajectory components were well-approximated as linear, providing justification for our choice of linear state model. However, cluster interpretability in some of these previous approaches^34,35^ may be limited by small sample sizes ($n < 100$). Further, given the paucity of available longitudinal data in research and clinical practice, it may be difficult to learn the underlying dynamics with modeling frameworks that may allow more degrees of freedom (i.e. kernel- or neural network-based approaches).

Specifically, MTM aims to map the variability structure in health trajectories, using state space models to model trajectories. We explore whether the trajectory variability in the data is captured through a limited number of “prototypical” state space models. We perform probabilistic clustering of trajectories using probabilistic mixture modeling where the individual mixture components correspond to those prototypical state space models. This is inherently an unsupervised learning process. However, as health trajectories contain information about the dynamics of disease progression, it is expected that clusters of trajectories governed by the corresponding group-level state space models relate to different disease progression stages. In comparison to previous trajectory modeling approaches (SuStaIn;^37–39^) that focus on stratifying disease subtypes based on cross-sectional data and require data from individuals who have progressed to later disease stages, MTM focuses on health trajectories and disease progression, targeting stratification of healthy or at-risk individuals. Further, MTM takes into account rate of decline (i.e. time between assessments) rather than simply event order^37^, allowing us to robustly stratify individuals at early stages based on disease progression compared to modeling approaches that require data from individuals at advanced disease stages to differentiate between dementia subtypes. Thus, MTM allows us to make inferences about an individual’s future cognitive health given their probabilistic assignment to a cluster based on their multimodal health trajectory. We demonstrate that model training on trajectories offers an advantage over training on baseline data alone; that is, MTM stratifies individuals more reliably into clusters when trained on longitudinal data. To formalize this, we derive an individualized index of cluster assignment and show that this index predicts conversion to AD more precisely than standard clinical data (grey matter atrophy, β-amyloid, cognitive scores).

Our findings provide the following main advances with strong potential for clinical translation. First, MTM reliably stratifies individuals to clinically meaningful clusters and allows us to derive an individualized index of cognitive health that predicts conversion to AD more reliably than standard clinical assessments (i.e. cognitive tests or MRI scans alone) with strong potential for reducing risk of misdiagnosis. Further, investigating individual variability in cognitive health and resilience remains a key challenge for understanding the underlying mechanisms of neurodegenerative disorders^40,41^. That is, why do some individuals show increased β-amyloid burden without experiencing grey matter loss^42,43^ or clinical cognitive symptoms^44–46^, or others experience grey matter loss without exhibiting memory symptoms^47–49^? MTM allows us to determine how individuals in different clusters vary in disease progression based on the combination of different markers. For example, for individuals in cluster A, β-amyloid burden does not significantly impact grey matter degeneration or memory decline. Individuals in cluster C have similar β-amyloid accumulation but higher grey matter atrophy compared to individuals in cluster B, suggesting that individuals in cluster C may have progressed to later neurodegeneration stages than individuals in cluster B. Thus, MTM offers a framework for precise individual patient stratification and individualized predictions of cognitive health early (i.e. at early or pre-symptomatic disease stages) with strong translational potential for clinical management and personalized interventions for improved patient outcomes.

Second, MTM harnesses the power of longitudinal multimodal data during training (i.e., learning relationships between biomarker and cognitive data over time) and generalizes to independent test datasets with missing data. In particular, MTM reliably stratifies individuals when tested with data from single assessments or cognitive trajectories alone. This has the potential to expedite clinical diagnosis from first assessment, reducing time to diagnosis-that currently varies between 6 and 18 months-and enhancing clinical management efficiency. Further, unsupervised modeling of non-invasive and low-cost data (e.g., cognitive tests) has strong potential to support early dementia prediction at scale from non-invasive testing, reducing patient burden, costs and health inequalities (e.g., due to limited access to MRI/PET scanners).

Third, MTM generalizes from research to clinical cohort data; that is, we validate our model by testing not only on research (ADNI) but also an independent clinical cohorts (MACC) despite missing data. Research cohort data may be subject to biases (e.g., selective volunteer demographics) while patient data tends to be more diverse and representative of the population at large. Validating MTM with data from patients across different settings (i.e., different MRI scanners, cognitive screening tools) and countries (USA, Singapore) provides evidence for interoperability with strong potential for reducing bias and enhancing the clinical utility of our approach.

Finally, our approach has strong potential to generalize to other non-invasive and cost- effective data (e.g., digital markers from wearable technologies) and dementia subtypes (extending beyond AD), enhancing the translational impact of MTM for early and precise stratification at pre-symptomatic stages. In the future, MTM-as an unsupervised AI-guided tool-could be optimized to make early dementia predictions from digital data and implemented in brain health checks (e.g. digital assessments at home) to select individuals at-risk for more extensive follow-up, making early dementia prediction scalable and cost-effective with potential impact for prevention and population health.

### Supplementary Information

Supplementary Information.

## Data Availability

Data and code used for the figures in this manuscript are publicly available at the University of Cambridge data repository: https://doi.org/10.17863/CAM.102058
