# Supplementary material for: Unsupervised multimodal modeling of cognitive and brain health trajectories for early dementia prediction
Source: Sci Rep. 2024 May 10;14:10755. doi: 10.1038/s41598-024-60914-w (PMC11087538; doi:10.1038/s41598-024-60914-w)
Supplement: Supplementary file 1 — Supplementary Information. [file 41598_2024_60914_MOESM1_ESM.pdf]

Supplementary Methods

Datasets

Two cohorts were considered for model training and testing: ADNI and MACC. Ethical approvals for ADNI were collected by the ADNI investigators and ethical approvals for MACC were collected by the Memory Ageing & Cognition Centre at the National University of Singapore. For the MACC study, ethical approval was obtained from the National Healthcare Group Domain-Specific Review Board and the study was conducted following the Declaration of Helsinki. Participants or their legal guardians provided written informed consent.

ADNI. We collated longitudinal data at regular 2-year intervals from 571 individuals in ADNI. For each time point, we collated the following data: grey matter density from the medial temporal cortex, β-amyloid PET, composite scores for ADNI-Mem related to performance in memory tasks [1] and ADNI-EF related to performance in executive function tasks [2], and scores from the Montreal Cognitive Assessment [MoCA; 3], the Alzheimer's Disease Assessment Scale [ADAS-13; 4], and the mini-mental state examination [MMSE; 5]. The exam date was used as the canonical time reference for each data instance, and it was at this date that clinical diagnoses were made and MoCA, ADAS-13, and MMSE tests were administered. Grey matter density was assigned to a data instance if the corresponding T1 image was collected within 6 months of the exam date. Trajectories were formed from data instances for each participant by looking for available data within a 6-month window of baseline + 2 years, baseline + 4 years, etc., where baseline corresponds to the first instance where cognitive scores, grey matter density, and β-amyloid were available for a person. Clinical diagnoses based on individuals' final assessments were as follows: cognitively normal (CN: 234), stable MCI (sMCI: 224), progressive MCI (pMCI: 19), and Alzheimer's disease (AD: 94). Patients were identified as pMCI if they progressed from MCI to AD within a period of 3 years, while sMCI patients remained diagnosed as MCI for the same period. For quality control, individuals whose diagnosis switched from AD to MCI were removed from the dataset. Additionally, individuals whose standardized grey matter density score increased by 25% or more from one visit to the next were removed from the dataset. Demographics (Table S1) at baseline were as follows: the average age was 72.3 years (± std. of 6.9), the average educational attainment was 16.4 years (± std. of

2.6), and 48% of participants were female. 40% of individuals were identified as APOE4 positive (i.e., they had one or two ε4 alleles) and the remainder were APOE4 negative.

Structural MRI scans (T1) for ADNI participants were acquired across ADNI sites as described online (see: `https://adni.loni.usc.edu/methods/documents/mri-protocols`). T1 scans were processed to extract grey matter density scores from medial temporal cortex following the method described by Giorgio et al [6]. T1 scans were pre-processed with SPM 12 [7] to segment them into grey matter, white matter, and cerebrospinal fluid. They were normalized to a study-specific template using the DARTEL toolbox [8] and then individual grey matter segmentation volumes were normalized to MNI space without modulation. At each voxel location, unmodulated values represent grey matter density. The images were then smoothed with a 3mm3 isotropic kernel and resliced to MNI resolution 1.5mm × 1.5mm × 1.5mm voxel size. We then calculated a previously-validated disease-specific biomarker using the processed images. This biomarker was formed by applying partial least squares regression [9, §12.5.2] with recursive feature elimination to create orthogonal features from T1-weighted MRI voxels having maximum covariance with MMSE score (in this way, the scores were also normalized at the voxel level, so values are often negative). In this context, recursive feature elimination was used to iteratively remove the least relevant voxels. As the resulting value is density- and not volume-based, differences in brain size do not impact the biomarker score.

PET imaging with the Florbetapir ([18F]AV45, FBP) radiotracer was performed at each ADNI site using methods as described in the respective protocols. FBP data were realigned and co-registration was performed to each participant's structural MRI using the mean of all frames. Cortical standardized uptake value ratios (SUVRs) were generated by averaging FBP retention in a standard group of ROIs defined by FreeSurfer v5.3 [10] and then dividing by the average uptake in the whole cerebellum. These SUVRs were converted to centiloid [11] scale with the transformation $x \mapsto 196.9 \cdot x - 196.03$.

MACC. We collated longitudinal data with help from the Memory Ageing & Cognition Centre at the National University of Singapore. Data was collected as part of both the Harmonization study [12]–[15] and the ABRI sub-study [16]–[18]. The MACC dataset consisted of 157 trajectories containing a single PET measurement (i.e. β-amyloid) and at least one MRI (i.e., grey matter density from medial temporal cortex), MoCA and MMSE scores. Trajectories were formed using available data from study visits:

baseline, year 2, year 4. Clinical diagnoses based on individuals' final available assessments were as follows: cognitively normal (CN: 36), mild MCI (50), moderate MCI (19), and Alzheimer's disease (AD: 55). Demographics at baseline were as follows: the average age was 73.0 years (± std. of 7.4), average educational attainment was 7.9 years (± std. of 4.8), and 55% of participants were female. 30.4% of individuals were APOE4 positive.

T1 MRI scans were acquired with a 3T Magnetom Trio, a Tim system (Siemens) with a 32-channel head coil, at Clinical Imaging Research Center of the National University of Singapore. Grey matter density scores were generated using the same method as for ADNI. PET imaging with the C-labelled Pittsburgh Compound-B ([11C]PiB) was also performed at the Clinical Imaging Research Center. Images were registered to the same space as used by the ADNI study, and regions of interest (ROI) templates for the cortical regions of the frontal, parietal, temporal, occipital, anterior and posterior cingulate, nucleus accumbens, and thalamus [19]. Detailed descriptions of the methodology can be found in Nai et al [20]. The SUVR values were converted to centiloid scale using the linear map $x \mapsto 100(x-1.009)/1.067$.

Model training

The models learned on ADNI were trained using standard 10-fold cross validation. The data is partitioned uniformly at random into 10 subsets of approximately equal size. For each subset i, $1 \leq i \leq 10$, we train a model using data from the 9 other subsets and predict on the ith subset. For each fold, we trained the MTM using the data from the 9 other folds ($n \approx 514$ trajectories) training on the remaining data ($n \approx 57$ trajectories).

Individual models were trained as follows. We start with a training dataset:

$$\mathcal{D} = \{(x^i_{1:T}, z^i_{1:T})\}_{1 \leq i \leq n_d}$$

consisting of paired sequences measurements $x^i_{1:T} = (x^i_1, x^i_2, \ldots, x^i_T)$ where $x^i_t \in \mathbb{R}^\ell$ and states $z^i_{1:T} = (z^i_1, z^i_2, \ldots, z^i_T)$ where $z^i_t \in \mathbb{R}^d$. In an abuse of notation, we allow the length of each sequence to depend on i. We adopt a mixture of linear gaussian state space models with n_c components, as described in (**??**)

and (**??**):

$$(1) \qquad p(z^i_{1:T}, x^i_{1:T}) = \sum_{c=1}^{n_c} \pi_c \delta_{\{c=c^i\}} \big(\eta_d(z_1; m_c, S_c) \prod_{t=2}^{T} \eta_d(z_t; z_{t-1}A_c, \Gamma_c) \prod_{t=1}^{T} \eta_\ell(x_t; z_t H_c, \Lambda_c)\big)$$

where $\pi_c \in \mathbb{R}^{n_c}_{\geq 0}$ governs the population-level cluster distribution so that $\sum_{c=1}^{n_c} \pi_c = 1$, $m_c \in \mathbb{R}^\ell$ and $S_c \in \mathbb{S}_\ell$ parameterise the initial state for cluster c, $A_c \in \mathbb{R}^{d\times d}$ and $\Gamma_c \in \mathbb{S}_d$ describe the dynamics of the states, and $H_c \in \mathbb{R}^{d\times\ell}$ and $\Lambda_c \in \mathbb{S}_\ell$ describe the relationship between the states and the measurements. Here, $\mathbb{S}_\ell$ denotes the set of valid $\ell \times \ell$ covariance matrices.

We performed expectation–maximization [EM; 21] with hard cluster assignment to train models. Each individual model was trained from 1000 different initializations of cluster assignment. The first initialization was the output of k-means applied to the initial state in each trajectory. Performing k-means over all hidden states was not possible, as trajectories varied in length. Specifically, we used the scikit-learn [22] instantiation of the k-means++ algorithm [23]. In the M-step, for each cluster, we used the trajectories assigned to the cluster and learned the model-specific parameters as follows. The mean mc and covariance Sc of the initial state were set to the empirical mean and covariance. The other cluster-specific parameters were learned with regularized linear regression. The global cluster assignment parameter π was set to the empirical incidence rate of each cluster. The E-step was then performed in the standard manner, assigning each trajectory to the cluster to which it was most likely to belong. Training ran for 1000 steps or until convergence. If none of the hard assignments changed in a given iteration, then EM has successfully completed. After completing training for each of the initializations, we selected the model having the highest expected complete data log likelihood:

$$(2) \qquad \sum_{i=1}^{n_d} \sum_{k=1}^{n_c} r_{ic} \log \pi_c + \sum_{i=1}^{n_d} \sum_{c=1}^{n_c} r_{ic} \log p\big(x^i_{1:T}, z^i_{1:T} | \Theta_c\big)$$

where $\Theta = \{m_c, S_c, A_c, \Gamma_c, H_c, \Lambda_c\}$ denotes the cluster-specific parameters for the cth cluster and r_{ic} is 1 when the ith datapoint is most likely to belong to the cth cluster and 0 otherwise.

Cluster demographics and profiling

Tables S4A and S4B summarize the cluster-wise demographics for ADNI and MACC clusters.

Ablation against an unsupervised model trained on baseline features alone

GMM-baseline. To test the advantage of modeling trajectories compared to cross-sectional snapshots, we trained a Gaussian mixture model on baseline data from each trajectory with scikit-learn [22] from a k-means initialization for each cross-validation fold (on the same folds as used to train the MTM). Labels were assigned to the clusters identified by each of the models in the same manner as for MTM clusters. The resulting assignments are referred to as GMM-baseline. Further, we compared cluster assignment for GMM-baseline against MTM trained on longitudinal data but tested on baseline data alone. The resulting assignments are referred to as MTM-baseline.

Survival Modeling with Cox Proportional Hazards Models

All Cox proportional hazards models [24] were trained with the lifelines package [25] using Breslow's estimator [26] for the baseline. In every case, the outcome of interest was an AD diagnosis during a person's trajectory, among persons not having an AD diagnosis at baseline. For the univariate analysis, 10-fold cross-validation was used with mild regularization, allowing the concordance index [27] to be calculated on heldout data.

Model marginalization for missing data

A single linear Gaussian state space model is jointly Gaussian in $(z_{1:T}, x_{1:T})$, allowing one to exactly and efficiently marginalize over missing variables and missing components of variables. For example, given a new participant having only measurement data, we can compute $p_c(x_{1:T}) := p(x_{1:T}|c)$ for each c to estimate cluster membership for this new participant as

$$p(c|x_{1:T}) \propto p_c(x_{1:T})p(c)$$

where the constant of proportionality depends on $x_{1:T}$ and we have

$$p_c(x_{1:T}) = \int p_c(x_{1:T}, z_{1:T}) dz_{1:T}.$$

Integrating over multivariate Gaussians can be done analytically. This process of marginalizing out unavailable or missing data can also be used during the training process, avoiding data imputation.

In particular, we derive a closed-form expression for a single component of the trajectory model. The joint distribution on the states given by:

$$p(z_{1:T}) = \eta_d(z_1; m, S) \prod_{t=2}^{T} \eta_d(z_t; z_{t-1}A, \Gamma)$$

may be re-expressed as:

$$p(z_{1:T}) = \eta_{dT}\left(\begin{bmatrix} z_1 \\ z_2 \\ z_3 \\ \vdots \\ z_T \end{bmatrix}; \begin{bmatrix} m \\ mA \\ mA^2 \\ \vdots \\ mA^{T-1} \end{bmatrix}, \begin{bmatrix} S & A'S & (A')^2 S & \cdots \\ SA & \Gamma + A'SA & A'(\Gamma + A'SA) & \\ SA^2 & (\Gamma + A'SA)A & \Gamma + A'(\Gamma + A'SA)A & \\ \vdots & & & \ddots \end{bmatrix}\right)$$

where each entry in the mean vector is a $d \times 1$ block and each entry in the covariance matrix is a $d \times d$ block. We use A' to denote the transpose of the matrix A to avoid any confusion between T and $\top$. If we define the diagonal elements as follows

$$\begin{aligned}
C_{11} &= S \\
C_{22} &= \Gamma + A'C_{11}A = \Gamma + A'SA \\
C_{33} &= \Gamma + A'C_{22}A = \Gamma + A'\Gamma A + (A')^2 SA^2 \\
C_{44} &= \Gamma + A'C_{33}A = \Gamma + A'\Gamma A + (A')^2 \Gamma A^2 + (A')^3 SA^3 \\
&\vdots \\
C_{jj} &= (A')^{j-1} SA^{j-1} + \textstyle\sum_{k=0}^{j-2} (A')^k \Gamma A^k
\end{aligned}$$

for $1 \leq j < T$, and for $i > j$, let $C_{ij} = C_{ii}A^{i-j}$ where $C_{ji} = C'_{ij}$, then we find that:

$$p(z_{1:T}) = \eta_{dT}\left(\begin{bmatrix} z_1 \\ z_2 \\ z_3 \\ \vdots \\ z_T \end{bmatrix}; \begin{bmatrix} m \\ mA \\ mA^2 \\ \vdots \\ mA^{T-1} \end{bmatrix}, \begin{bmatrix} C_{11} & C_{12} & C_{13} & \cdots & C_{1T} \\ C_{21} & C_{22} & C_{23} & \cdots & C_{2T} \\ C_{31} & C_{32} & C_{33} & \cdots & C_{3T} \\ \vdots & \vdots & \vdots & \ddots & \vdots \\ C_{T1} & C_{T2} & C_{T3} & \cdots & C_{TT} \end{bmatrix}\right).$$

This gives us a closed form expression for the joint Gaussian distribution of the hidden states. We can then use the rules for Gaussian distributions to determine the joint distribution:

$$p(x_{1:T}, z_{1:T}) = \eta_{(d+\ell)T}\left(\begin{bmatrix} z_1 \\ \vdots \\ z_T \\ x_1 \\ \vdots \\ x_T \end{bmatrix}; \begin{bmatrix} m \\ \vdots \\ mA^{T-1} \\ mH \\ \vdots \\ mA^{T-1}H \end{bmatrix}, \begin{bmatrix} C_{11} & \cdots & C_{1T} & C_{11}H & \cdots & C_{1T}H \\ \vdots & \ddots & \vdots & \vdots & \ddots & \vdots \\ C_{T1} & \cdots & C_{TT} & C_{T1}H & \cdots & C_{TT}H \\ H'C_{11} & \cdots & H'C_{1T} & \Lambda + H'C_{11}H & \cdots & H'C_{1T}H \\ \vdots & \ddots & \vdots & \vdots & \ddots & \vdots \\ H'C_{T1} & \cdots & H'C_{TT} & H'C_{T1}H & \cdots & \Lambda + H'C_{TT}H \end{bmatrix}\right).$$

From here, marginalising out hidden or missing random variables can be done by removing minors in the covariance matrix and the corresponding elements in the mean vector. For example, we have the joint distribution of the measurements:

$$p(x_{1:T}) = \eta_{\ell T}\left(\begin{bmatrix} x_1 \\ x_2 \\ x_3 \\ \vdots \\ x_T \end{bmatrix}; \begin{bmatrix} mH \\ mAH \\ mA^2H \\ \vdots \\ mA^{T-1}H \end{bmatrix}, \begin{bmatrix} \Lambda + H'C_{11}H & H'C_{12}H & H'C_{13}H & \cdots & H'C_{1T}H \\ H'C_{21}H & \Lambda + H'C_{22}H & H'C_{23}H & \cdots & H'C_{2T}H \\ H'C_{31}H & H'C_{32}H & \Lambda + H'C_{33}H & \cdots & H'C_{3T}H \\ \vdots & \vdots & \vdots & \ddots & \vdots \\ H'C_{T1}H & H'C_{T2}H & H'C_{T3}H & \cdots & \Lambda + H'C_{TT}H \end{bmatrix}\right).$$

MTM with nonlinear specifications

Linear Gaussian dynamics are well understood and support analytic marginalization for training and predicting in the presence of missing data. However, in many cases, this formulation may prove restrictive for practical applications. For example, a linear model does not allow grey matter deterioration to accelerate at any point during the progression of dementia, nor does it allow for some biomarkers like β-amyloid to stop accumulating after reaching maximum saturation level in the brain. Modeling with nonlinear dynamics allow us to capture such non-linear relationships. In the first instance, we adopt a hybrid framework that maintains the linear Gaussian state model but learns nonlinear class-conditional measurement models $p_c(x_t|z_t) = \eta(x_t; f(z_t), \Lambda_c)$ where f is a k-NN regressor on training data. Our choice of hard assignment in the E step of EM makes learning the cluster-specific dynamics in the M step straightforward. In the reported results, we select k, the number of neighbors, with cross-validation (on 5, 10, or 15 neighbors). We report results in Fig. S3 and Table S3.

Data and Code availability

Data and code used for the figures in this manuscript will be made publicly available in the University of Cambridge data repository.

Supplementary References

[1] P. K. Crane, A. Carle, L. E. Gibbons, P. Insel, R. S. Mackin, A. Gross, *et al.*, "Development and assessment of a composite score for memory in the Alzheimer's disease neuroimaging initiative (ADNI)," *Brain Imaging Behav.*, vol. 6, no. 4, pp. 502–516, 2012.

[2] L. E. Gibbons, A. C. Carle, R. S. Mackin, D. Harvey, S. Mukherjee, P. Insel, *et al.*, "A composite score for executive functioning, validated in Alzheimer's disease neuroimaging initiative (ADNI) participants with baseline mild cognitive impairment," *Brain Imaging Behav.*, vol. 6, no. 4, pp. 517–527, 2012.

[3] Z. S. Nasreddine, N. A. Phillips, V. Bédirian, S. Charbonneau, V. Whitehead, I. Collin, *et al.*, "The Montreal cognitive assessment, MoCA: A brief screening tool for mild cognitive impairment," *J. Am. Geriatr. Soc.*, vol. 53, no. 4, pp. 695–699, 2005.

[4] R. Mohs, D. Knopman, P. R.C., S. Ferris, E. C., G. M., *et al.*, "Development of cognitive instruments for use in clinical trials of antidementia drugs: Additions to the Alzheimer's disease assessment scale that broaden its scope," *Alzheimer Dis. Assoc. Disord.*, vol. 11, 1997.

[5] M. Folstein, S. Folstein, and P. McHugh, ""Mini-mental state": A practical method for grading the cognitive state of patients for the clinician," *J. Psychiatr. Res.*, vol. 12, no. 3, pp. 189–198, 1975.

[6] J. Giorgio, S. M. Landau, W. J. Jagust, P. Tino, and Z. Kourtzi, "Modelling prognostic trajectories of cognitive decline due to Alzheimer's disease," *NeuroImage Clin.*, vol. 26, 2020.

[7] W. Penny, K. Friston, J. Ashburner, S. Kiebel, and T. Nichols, *Statistical Parametric Mapping: The Analysis of Functional Brain Images*. Academic Press, 2006.

[8] J. Ashburner, "A fast diffeomorphic image registration algorithm," *NeuroImage*, vol. 38, no. 1, pp. 95–113, 2007.

[9] K. P. Murphy, *Machine Learning: A Probabilistic Perspective*. Cambridge, U.S.A.: MIT Press, 2012.

[10] B. Fischl, "Freesurfer," *NeuroImage*, vol. 62, pp. 774–781, 2012.

[11] W. E. Klunk, R. A. Koeppe, J. C. Price, T. L. Benzinger, M. D. Devous, W. J. Jagust, *et al.*, "The centiloid project standardizing quantitative amyloid plaque estimation by PET," *Alzheimers Dement.*, vol. 11, no. 1, pp. 1–15, 2015.

[12] B. Gyanwali, C. Tan, J. Petr, L. Escobosa, H. Vrooman, C. Chen, *et al.*, "Arterial spin-labeling parameters and their associations with risk factors, cerebral small-vessel disease, and etiologic subtypes of cognitive impairment and dementia," *AJNR Am. J. Neuroradiol.*, vol. 43, no. 10, pp. 1418–1423, 2022.

[13] L.-Y. Wu, C. N. Kan, I. K. Cheah, J. R. Chong, X. Xu, H. Vrooman, *et al.*, "Low plasma ergothioneine predicts cognitive and functional decline in an elderly cohort attending memory clinics," *Antioxidants*, vol. 11, no. 9, 2022.

[14] C. N. Kan, J. Cano, X. Zhao, Z. Ismail, C. L.-H. Chen, and X. Xu, "Prevalence, clinical correlates, cognitive trajectories, and dementia risk associated with mild behavioral impairment in asians," *J. Clin. Psychiatry*, vol. 83, no. 3, 2022.

[15] S. S Hilal, C. Cheung, T. Y. Wong, L Schmetterer, and C. Chen, "Retinal parameters, cortical cerebral microinfarcts, and their interaction with cognitive impairment," *Int. J. Stroke*, vol. 18, no. 1, pp. 70–77, 2023.

[16] J. R. Chong, N. J. Ashton, T. K. Karikari, T. Tanaka, F. N. Saridin, A. Reilhac, *et al.*, "Plasma p-tau181 to aβ42 ratio is associated with brain amyloid burden and hippocampal atrophy in an asian cohort of Alzheimer's disease patients with concomitant cerebrovascular disease," *Alzheimers Dement.*, vol. 17, no. 10, pp. 1649–1662, 2021.

[17] M. J. R. Lim, J. Tan, B. Gyanwali, T. Tanaka, A. Reilhac, H. A. Vrooman, *et al.*, "The associations between intracranial stenosis, brain amyloid-beta, and cognition in a memory clinic sample," *Alzheimer Dis. Assoc. Disord.*, vol. 36, no. 4, 2022.

[18] F. N. Saridin, K. A. Chew, A. Reilhac, B. Gyanwali, S. G. Villaraza, T. Tanaka, *et al.*, "Cerebrovascular disease in suspected non-Alzheimer's pathophysiology and cognitive decline over time," *Eur. J. Neurol.*, vol. 29, pp. 1922–1929, 2022.

[19] M. Tanveer, B. Richhariya, R. U. Khan, A. H. Rashid, P. Khanna, M. Prasad, *et al.*, “Machine learning techniques for the diagnosis of Alzheimer’s disease: A review,” *ACM Trans. Multimed. Comput. Commun. Appl.*, vol. 16, no. 1s, 2020.

[20] Y. Nai, Y. Tay, T. Tanaka, C. P. Chen, E. G. Robins, and A. Reilhac, “Comparison of three automated approaches for classification of amyloid-pet images,” *Neuroinformatics*, vol. 20, pp. 1065–1075, 2022.

[21] A. Dempster, N. Laird, and D. B. Rubin, “Maximum likelihood from incomplete data via the EM algorithm,” *J. Roy. Stat. Soc. Ser. B (Stat. Methodol.)*, vol. 39, no. 1, pp. 1–38, 1977.

[22] F. Pedregosa, G. Varoquaux, A. Gramfort, V. Michel, B. Thirion, O. Grisel, *et al.*, “Scikit-learn: Machine learning in Python,” *J. Mach. Learn. Res.*, vol. 12, pp. 2825–2830, 2011.

[23] D. Arthur and S. Vassilvitskii, “K-means++: The advantages of careful seeding,” in *Proc. ACM-SIAM Symp. Discret. Algoritm.*, 2007.

[24] D. R. Cox, “Regression models and life-tables,” *J. Roy. Stat. Soc. Ser. B (Stat. Methodol.)*, vol. 34, no. 2, pp. 187–220, 1972.

[25] C. Davidson-Pilon, “Lifelines: Survival analysis in Python,” *J. Open Source Softw.*, vol. 4, no. 40, 2019.

[26] N. E. Breslow, “Analysis of survival data under the proportional hazards model,” *Int. Stat. Rev.*, vol. 43, no. 1, pp. 45–57, 1975.

[27] F. E. Harrell, K. L. Lee, and D. B. Mark, “Multivariable prognostic models: Issues in developing models, evaluating assumptions and adequacy, and measuring and reducing errors,” *Stat. Med.*, vol. 15, no. 4, pp. 361–387, 1996.

Supplementary Figures

Figure S1. Cluster assignment stability across cross-validation runs. For each trajectory assigned to a given cluster during cross-validated testing, we determine the number of training runs for which the trajectory was assigned to that cluster. Note that for 10-fold cross-validation, each trajectory is in a test set once and in a training set 9 times, resulting in values ranging from 0 to 9. **A**. Histogram for cluster A. **B**. Histogram for cluster B. **C**. Histogram for cluster C. **D**. Histogram for cluster D. All histograms concentrate on higher values (ranging from 7 to 9), indicating that most individual trajectories remain in the same cluster across cross-validation runs.

Figure S2. Model selection. A. Expected complete data log likelihood versus the number of clusters for the ADNI dataset. **B.** Bayesian Information Criterion [BIC; 1] versus the number of clusters for ADNI. **C.** Expected complete data log likelihood versus the number of clusters calculated on the MACC dataset for models trained on ADNI. **D.** BIC versus the number of clusters calculated on the MACC dataset for models trained on ADNI.

Figure S3. Progression of states over time for MTM with nonlinear specifications. A. Progression of states over time illustrating the relationship between state variables (grey matter density, β-amyloid burden (centiloid) per cluster, for 4-cluster MTM with linear Gaussian state dynamics and a nonlinear, Gaussian measurement model. **B.** Progression of states over time for 4-cluster MTM with a nonlinear, Gaussian state model and measurement model.

Figure S4. Cluster assignment by clinical outcome. A. Pie charts indicating clinical outcome and break down into cluster assignment indicating the frequency of each cluster given clinical outcome for ADNI. **B.** The same break down for MACC.

Figure S1

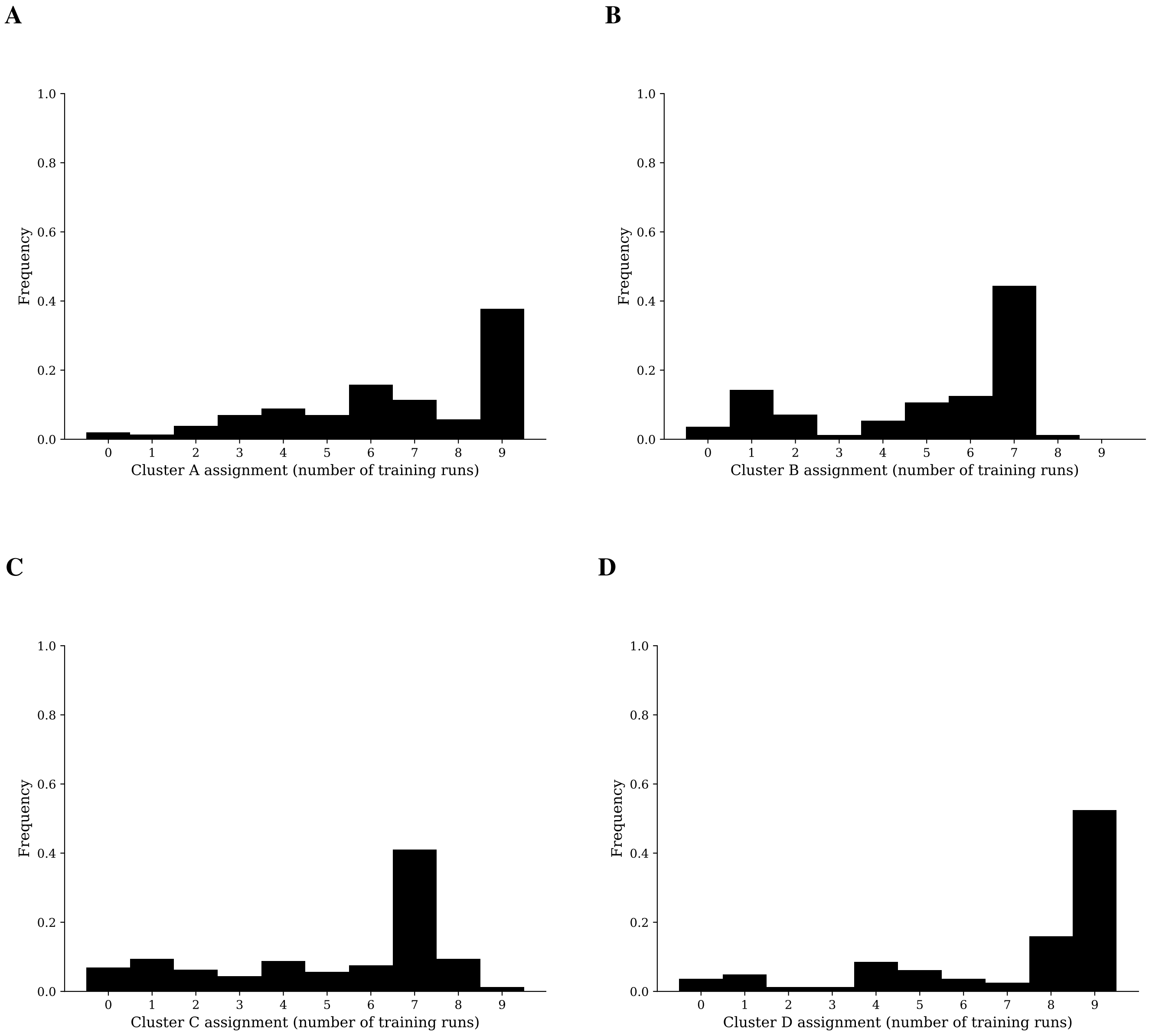
A
B
C
D
Frequency
1.0
0.8
0.6
0.4
0.2
0.0
0 1 2 3 4 5 6 7 8 9
Cluster A assignment (number of training runs)
Cluster B assignment (number of training runs)
Cluster C assignment (number of training runs)
Cluster D assignment (number of training runs)

Figure S2

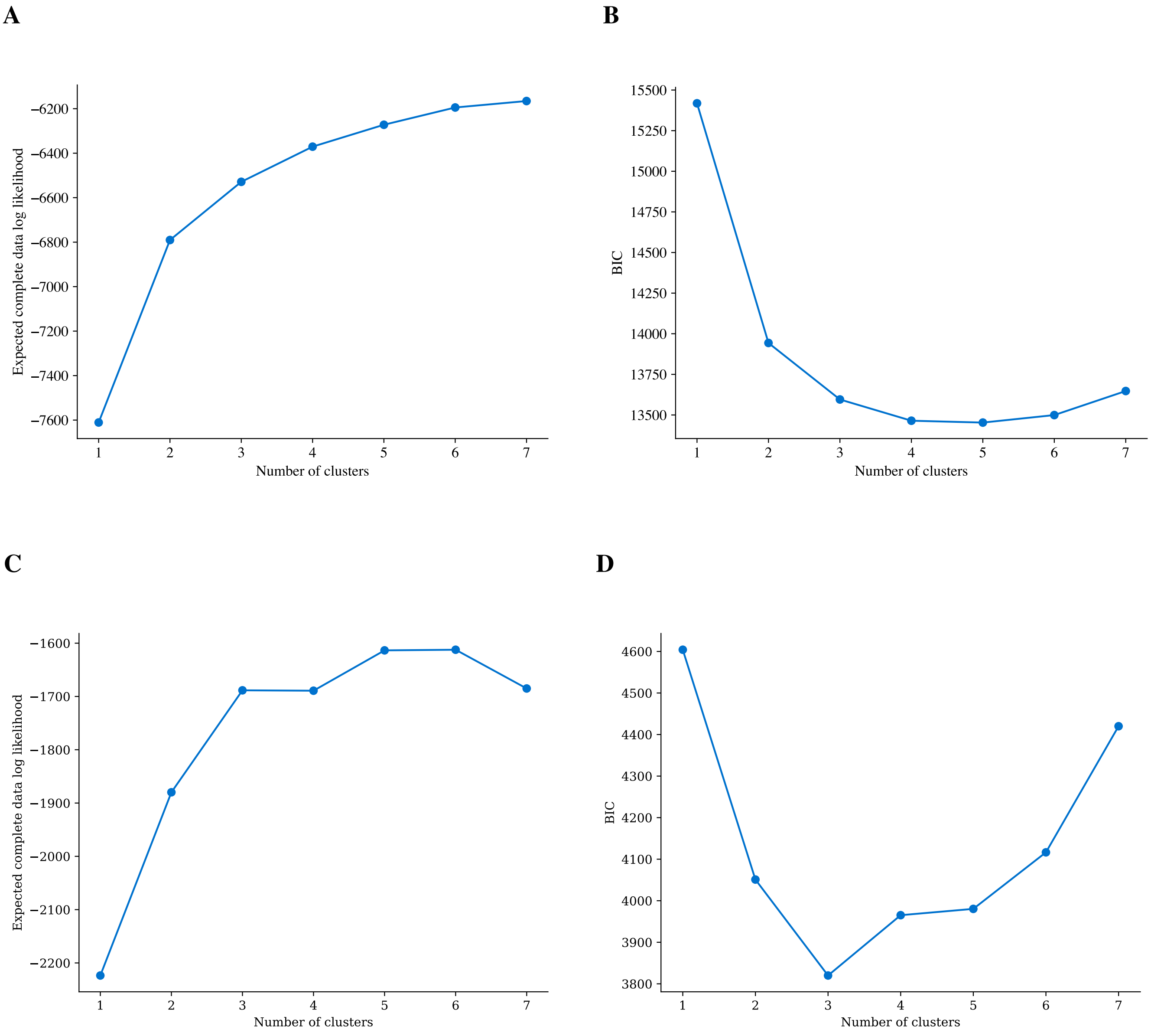
A
Expected complete data log likelihood
−6200
−6400
−6600
−6800
−7000
−7200
−7400
−7600
1 2 3 4 5 6 7
Number of clusters
B
BIC
15500
15250
15000
14750
14500
14250
14000
13750
13500
1 2 3 4 5 6 7
Number of clusters
C
Expected complete data log likelihood
−1600
−1700
−1800
−1900
−2000
−2100
−2200
1 2 3 4 5 6 7
Number of clusters
D
BIC
4600
4500
4400
4300
4200
4100
4000
3900
3800
1 2 3 4 5 6 7
Number of clusters

Figure S3

A

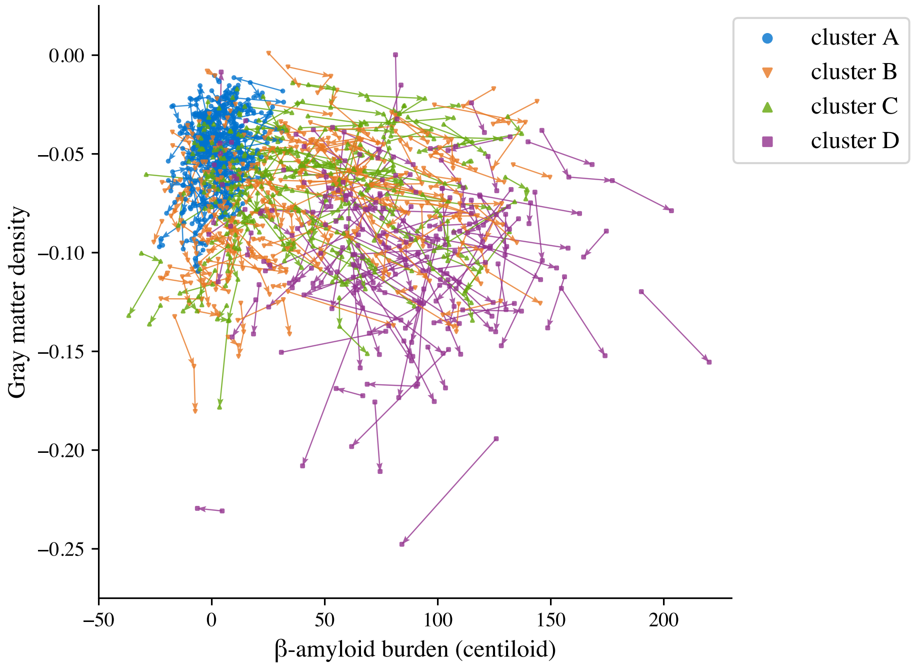
cluster A
cluster B
cluster C
cluster D
Gray matter density
0.00
−0.05
−0.10
−0.15
−0.20
−0.25
−50
0
50
100
150
200
β-amyloid burden (centiloid)

B

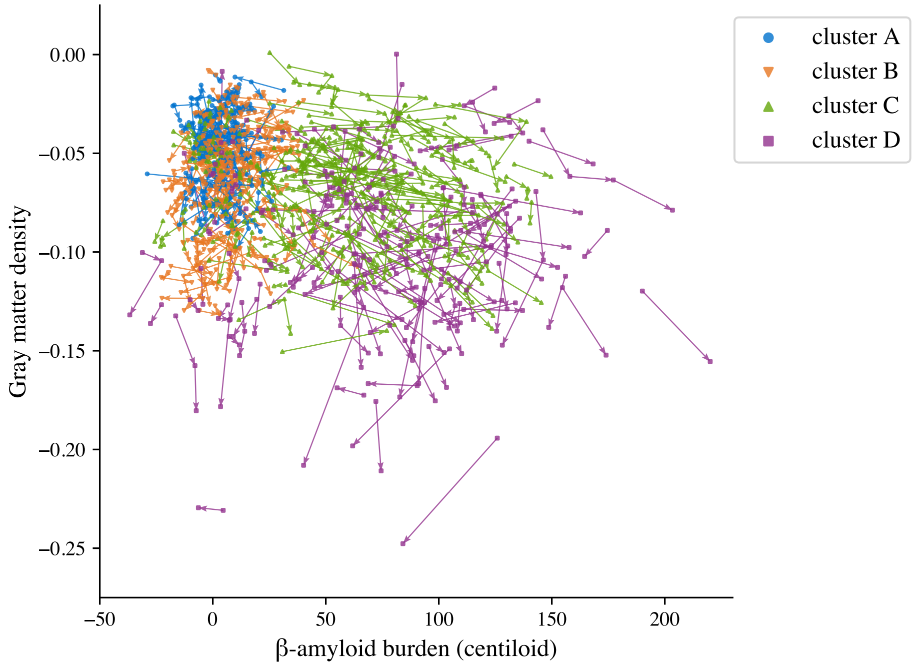
cluster A
cluster B
cluster C
cluster D
Gray matter density
0.00
−0.05
−0.10
−0.15
−0.20
−0.25
−50
0
50
100
150
200
β-amyloid burden (centiloid)

Figure S4

A

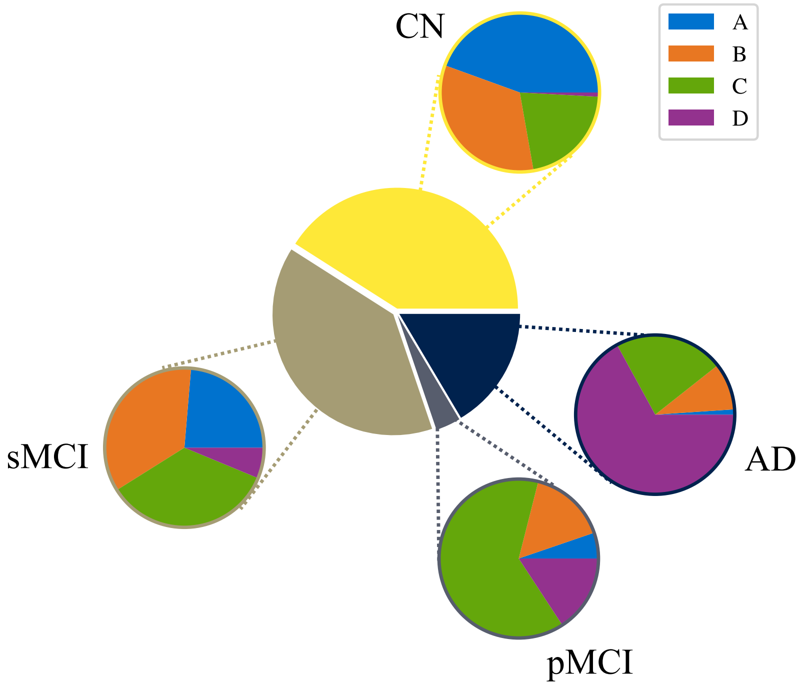

B

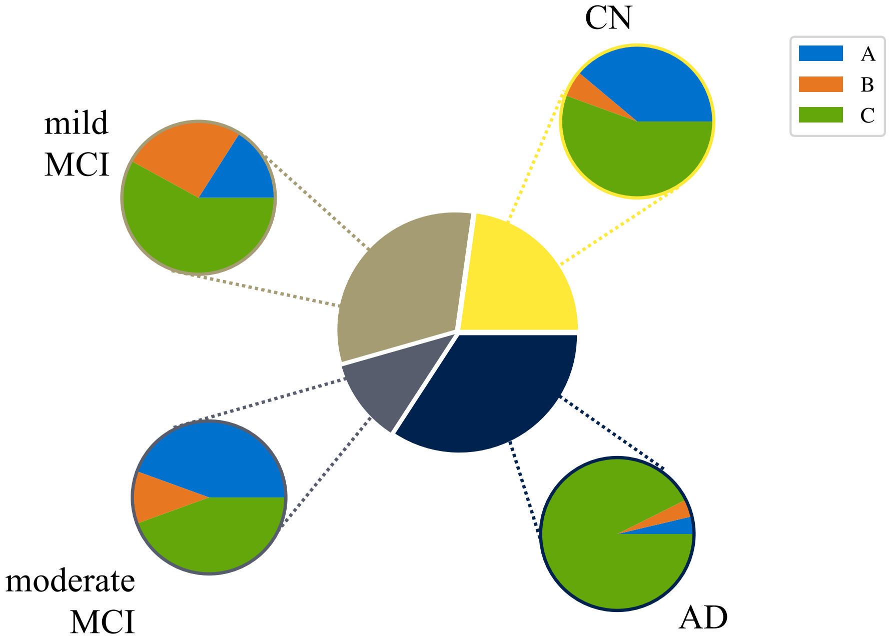

Table S1

Demographics and genetic marker (apoe4) per cluster. A. Cluster-wise averages for 4-cluster MTM on ADNI. **B.** Cluster-wise averages for 3-cluster MTM on MACC.

A

cluster	age	female	education	apoe4 pos.
A	70.6	48%	16.7	20%
B	71.6	51%	16.2	44%
C	75.7	44%	16.5	41%
D	75.0	46%	15.8	68%

B

cluster	age	female	education	apoe4 pos.
A	73.6	34%	8.8	22%
B	75.0	21%	8.7	16%
C	72.4	67%	7.4	36%

Table S2

Clinical diagnosis per cluster for MTM training folds. A. Cluster assignment frequencies for each cross-validated training run using ADNI data. Note that while the test datasets in cross-validation partition the dataset, the training datasets do not: any two runs share 8/9ths of their training data (i.e. training data across folds are not independent). See Table S4A column I for comparison. **B.** Clinical outcome frequencies per cluster on each of the ADNI training sets. See Table S4B column I for comparison.

A

cluster	run 1	run 2	run 3	run 4	run 5	run 6	run 7	run 8	run 9	run 10	**avg.**	std. err.
A	32%	21%	22%	30%	31%	30%	22%	33%	26%	24%	**27%**	1.4%
B	29%	35%	45%	32%	30%	26%	22%	28%	30%	29%	**30%**	2.0%
C	32%	26%	21%	28%	28%	32%	40%	22%	29%	30%	**29%**	1.7%
D	8%	18%	11%	11%	11%	12%	16%	17%	15%	17%	**14%**	1.1%

B

cluster	diagnosis	run 1	run 2	run 3	run 4	run 5	run 6	run 7	run 8	run 9	run 10	**avg.**	std. err.
A	CN	60%	73%	76%	59%	61%	56%	72%	63%	60%	64%	**64%**	2.2%
	sMCI	40%	27%	24%	40%	38%	44%	28%	37%	40%	36%	**35%**	2.2%
	pMCI	0%	0%	0%	0%	1%	0%	0%	0%	0%	0%	**0%**	0.1%
	AD	0%	0%	0%	1%	1%	0%	0%	0%	0%	0%	**0%**	0.1%
B	CN	49%	39%	42%	50%	45%	59%	47%	42%	50%	47%	**47%**	1.8%
	sMCI	45%	53%	51%	43%	48%	39%	41%	46%	46%	48%	**46%**	1.3%
	pMCI	2%	3%	3%	3%	3%	1%	6%	4%	1%	2%	**3%**	0.5%
	AD	4%	4%	5%	4%	4%	2%	5%	8%	3%	3%	**4%**	0.6%
C	CN	23%	42%	25%	27%	28%	25%	39%	40%	34%	35%	**32%**	2.2%
	sMCI	45%	43%	45%	45%	46%	48%	49%	41%	43%	47%	**45%**	0.7%
	pMCI	8%	6%	9%	8%	7%	8%	3%	4%	8%	8%	**7%**	0.6%
	AD	23%	9%	20%	20%	19%	19%	9%	15%	15%	11%	**16%**	1.6%
D	CN	3%	7%	0%	2%	0%	2%	4%	5%	4%	6%	**3%**	0.7%
	sMCI	8%	21%	12%	11%	10%	8%	14%	26%	17%	18%	**14%**	1.9%
	pMCI	0%	4%	2%	0%	2%	3%	5%	6%	5%	4%	**3%**	0.7%
	AD	90%	68%	86%	87%	88%	87%	77%	64%	74%	72%	**79%**	3.0%

Table S3

Clinical diagnosis per cluster for MTM with nonlinear specifications on ADNI. A. Cluster assignment frequencies and per-cluster clinical outcome frequencies for 4-cluster MTM with linear Gaussian state dynamics and a nonlinear, Gaussian measurement model. **B.** Same results for 4-cluster MTM with a nonlinear, Gaussian state model and measurement model. (See also Fig. S3.)

A

		within cluster			
cluster	prevalence	CN	sMCI	pMCI	AD
A	30%	61%	39%	0%	0%
B	28%	35%	56%	4%	5%
C	24%	50%	35%	5%	10%
D	19%	7%	20%	6%	68%

B

		within cluster			
cluster	prevalence	CN	sMCI	pMCI	AD
A	19%	78%	22%	0%	0%
B	27%	44%	53%	1%	2%
C	32%	40%	48%	4%	8%
D	21%	6%	25%	7%	62%

Table S4

Cluster assignment for 4-cluster MTM trained and tested on ADNI. A. Cluster assignment frequencies from cross-validated predictions on: I. complete trajectories, II. trajectories of cognitive data alone, III. all features from the first assessment (baseline), IV. cognitive scores from the first assessment, V. all scores from the final assessment (this is the assessment to which clinical labels are associated), and VI. Cognitive scores from the final assessment. **B.** Clinical outcome frequencies per cluster. Columns as in A.

A

	trajectories		1st assessment		last assessment	
	I.	II.	III.	IV.	V.	VI.
cluster	all	cognitive only	all	cognitive only	all	cognitive only
A	28%	34%	31%	34%	29%	33%
B	30%	29%	32%	35%	28%	31%
C	28%	25%	30%	25%	27%	23%
D	14%	12%	8%	7%	17%	14%

B

		trajectories		1st assessment		last assessment	
		I.	II.	III.	IV.	V.	VI.
cluster	diagnosis	all	cognitive only	all	cognitive only	all	cognitive only
A	CN	65%	65%	64%	61%	63%	66%
	sMCI	33%	35%	35%	34%	36%	33%
	pMCI	1%	1%	1%	1%	1%	1%
	AD	1%	0%	1%	4%	1%	0%
B	CN	46%	46%	41%	42%	48%	44%
	sMCI	47%	47%	46%	43%	46%	49%
	pMCI	2%	3%	2%	5%	2%	3%
	AD	5%	4%	11%	11%	5%	4%
C	CN	31%	23%	28%	24%	34%	23%
	sMCI	48%	51%	42%	48%	48%	52%
	pMCI	8%	6%	7%	3%	7%	7%
	AD	13%	21%	23%	24%	11%	18%
D	CN	2%	0%	4%	0%	4%	3%
	sMCI	17%	10%	21%	13%	21%	10%
	pMCI	4%	7%	4%	5%	4%	5%
	AD	77%	83%	71%	82%	71%	82%

Table S5

Relating MTM to GMM. A. Cluster assignment frequencies using I. MTM tested on trajectories, II. MTM tested on baseline data alone, III. GMM model tested on baseline ADNI data. Note that while II. and III. are tested on baseline feature data, MTM (II) is trained using longitudinal data, while GMM (III) is trained on baseline data alone. **B.** Clinical outcome frequencies per cluster. Columns as in A. (For both A and B, columns I & II match columns I & III in Table S4, respectively.)

A

cluster	I. MTM	II. MTM (baseline)	III. GMM (baseline)
A	28%	31%	45%
B	30%	32%	33%
C	28%	30%	14%
D	14%	8%	8%

B

cluster	diagnosis	I. MTM	II. MTM (baseline)	III. GMM (baseline)
A	CN	65%	64%	56%
	sMCI	33%	35%	42%
	pMCI	1%	1%	0%
	AD	1%	1%	1%
B	CN	46%	41%	40%
	sMCI	47%	46%	43%
	pMCI	2%	2%	3%
	AD	5%	11%	14%
C	CN	31%	28%	15%
	sMCI	48%	42%	31%
	pMCI	8%	7%	10%
	AD	13%	23%	45%
D	CN	2%	4%	2%
	sMCI	17%	21%	19%
	pMCI	4%	4%	12%
	AD	77%	71%	67%

Table S6

Cluster assignment for 3-cluster MTM tested on MACC. A. Cluster assignment frequencies using a model trained on ADNI to predict on MACC: I. complete trajectories, II. trajectories of cognitive data only, and III. all features from the final assessment. **B.** Clinical outcome frequencies per cluster. Columns as in A.

A

cluster	I. trajectories	II. cognitive only	III. single assessment
A	20%	39%	36%
B	12%	30%	22%
C	68%	31%	42%

B

cluster	diagnosis	I. trajectories	II. cognitive only	III. single assessment
A	CN	44%	48%	38%
	mild MCI	25%	40%	31%
	moderate MCI	25%	10%	16%
	AD	6%	2%	14%
B	CN	11%	11%	3%
	mild MCI	68%	40%	59%
	moderate MCI	11%	21%	21%
	AD	11%	28%	18%
C	CN	19%	2%	19%
	mild MCI	27%	12%	18%
	moderate MCI	8%	4%	3%
	AD	47%	82%	60%
